# Case report: Managing multisystem inflammatory syndrome in children (MIS-C) in Lao People's Democratic Republic, a success story

**DOI:** 10.3389/fped.2023.981880

**Published:** 2023-02-15

**Authors:** Vannida Douangboupha, Kouyang Nhiacha, Bounloth Sodaluck, Daosavanh Thepmixay, Kristina M. Krohn

**Affiliations:** ^1^ Pediatric Infectious Disease Ward, Mahosot Hospital, Vientiane Capital, Laos; ^2^ Pediatric Intensive Care and Cardiology Unit, Children's Hospital, Vientiane Capital, Laos; ^3^ Pediatric Residency Training Program, University of Health Science, Vientiane Capital, Laos; ^4^ Department of Internal Medicine and Department of Pediatrics, University of Minnesota, Minneapolis, MN, United States

**Keywords:** MIS-C multisystem inflammatory syndrome in children, low- and lower-middle-income countries, children, hospitalized, low resource areas, COVID-19, SARS-CoV-2

## Abstract

**Introduction:**

Multisystem inflammatory syndrome in children (MIS-C) is believed to be one of the most important life-threatening complications of COVID-19 infection among children. In any setting, early recognition, investigations, and management of MIS-C is crucial, but it is particularly difficult in resource-limited settings (RLS). This is the first case report of MIS-C in Lao People's Democratic Republic (Lao PDR) that was promptly recognized, treated, and resulted in full recovery with no known complications despite the resource limitations.

**Case presentation:**

A healthy 9-year-old boy presented to a central teaching hospital fulfilling the World Health's Organization's MIS-C criteria. The patient had never received a COVID-19 vaccine and had a history of COVID-19 contact. The diagnosis was based upon the history, changes in the patient's clinical status, and response to treatment and negative testing and response to treatment for alternative diagnoses. Despite management challenges relating to limited access to an intensive care bed and the high cost of IVIG; the patient received a full course of treatment and appropriate follow-up cares post discharge. There were several aspects to this case that may not hold true for other children in Lao PDR. First, the family lived in the capital city, close to the central hospitals. Second, the family was able to afford repeated visits to private clinics, and the cost of IVIG, and other treatments. Third, the physicians involved in his care promptly recognized a new diagnosis.

**Conclusions:**

MIS-C is a rare but life-threatening complication of COVID-19 infection among children. The management of MIS-C requires early recognition, investigations, and interventions which may be difficult to access, cost-prohibitive, and further increase demand on healthcare services that are already limited in RLS. Nevertheless, clinicians must consider means for improving access, determine which tests and interventions are worth the cost, and establishing local clinical guidelines for working within resource constraints while awaiting additional assistance from local and international public health systems. Additionally, using COVID-19 vaccination to prevent MIS-C and its complication for children may be cost-effective.

## Introduction

As of December 15th, 2022, there were 646,740,524 confirmed COVID-19 cases and 6,637,512 total deaths from COVID-19 worldwide (1). As of December 15, 2022, Lao People's Democratic Republic (Lao PDR) reported 217,304 cases and 746 deaths (2). Of the total reported cases, 48% (104,938/217,304) were in those less-than-16-years-old (2). Eleven-to-15-year-olds accounted for 61% of children infected (63,630/104,938) followed by 5-to-10-year-olds (22%, 23,364/104,938), < 2 (9%, 9,780/104,938), and 2-to-5-year-olds (8%, 8,164/104,938) (2). The total number of deaths in children under 16 years old in Lao PDR was 18 (2).

Globally multisystem inflammatory syndrome in children (MIS-C) is a life-threatening inflammatory response (3) resulting from COVID-19 (4–6). It is caused by an abnormal immune response to the SARS-CoV-2 virus (7). It presents as a post-infectious complication of the virus rather than acute infection (8). Characteristics of MIS-C include persistent fever, systemic hyperinflammation, and multisystem organ dysfunction (9). Severe MIS-C patients usually have significant multisystem damage, including cardiac involvement and shock (8). MIS-C is rare and arose in less than 1% of confirmed SARS-CoV-2 cases in children with disproportionately lower reported cases in Asian children (8). There was a higher risk among males (7), older children (8–11-years-old) (8), and children with comorbidities (obesity and asthma) (8). The reasons why MIS-C affects different populations of children at different rates remains unclear. It may be due to different rates of exposure to SARS-CoV-2 in different populations. MIS-C is usually curable and/or transient, but its cardiac manifestations are immense and life-threatening (9).

Due to limited knowledge and experience with MIS-C, the wide spectrum of clinical presentations, and limited resources, families may present late, and diagnosis and treatment may be delayed in resource-limited settings (RLS) (10, 11). Many challenges including access to health care facilities and the availability of costly immunomodulatory drugs may be more difficult in RLS (12). Limited laboratory and radiologic studies in RLS are additional challenges for prompt diagnosis, appropriate follow-up, and appropriately excluding other diagnoses. These challenges may affect the morbidity and mortality of MIS-C patients in Lao PDR.

We report the first known MIS-C case in Lao PDR, a 9-year-old previously healthy boy with an 8-day-history of fever, 5-to-6-days of gastrointestinal symptoms and anorexia, 3-days of mucocutaneous symptoms and lethargy which rapidly improved after IVIG administration. This case report demonstrated that prompt recognition, diagnosis, and treatment can result in positive outcomes, even in RLS. This case also highlights what resources were available to this child but may not be available to other children in Lao PDR.

## Case presentation

### Patient information

A 9-year-old ethnically Lao boy was referred from a private clinic to a central hospital's emergency department (ED) in Vientiane, the capital city of Lao PDR, on December 20th, 2021, with a typical presentation of MIS-C. He had an 8-day-history of fever and a 5-day-history of abdominal pain in the setting of having family members recently test positive by PCR for COVID-19 on November 23rd, 2021.

**Figure 1:**
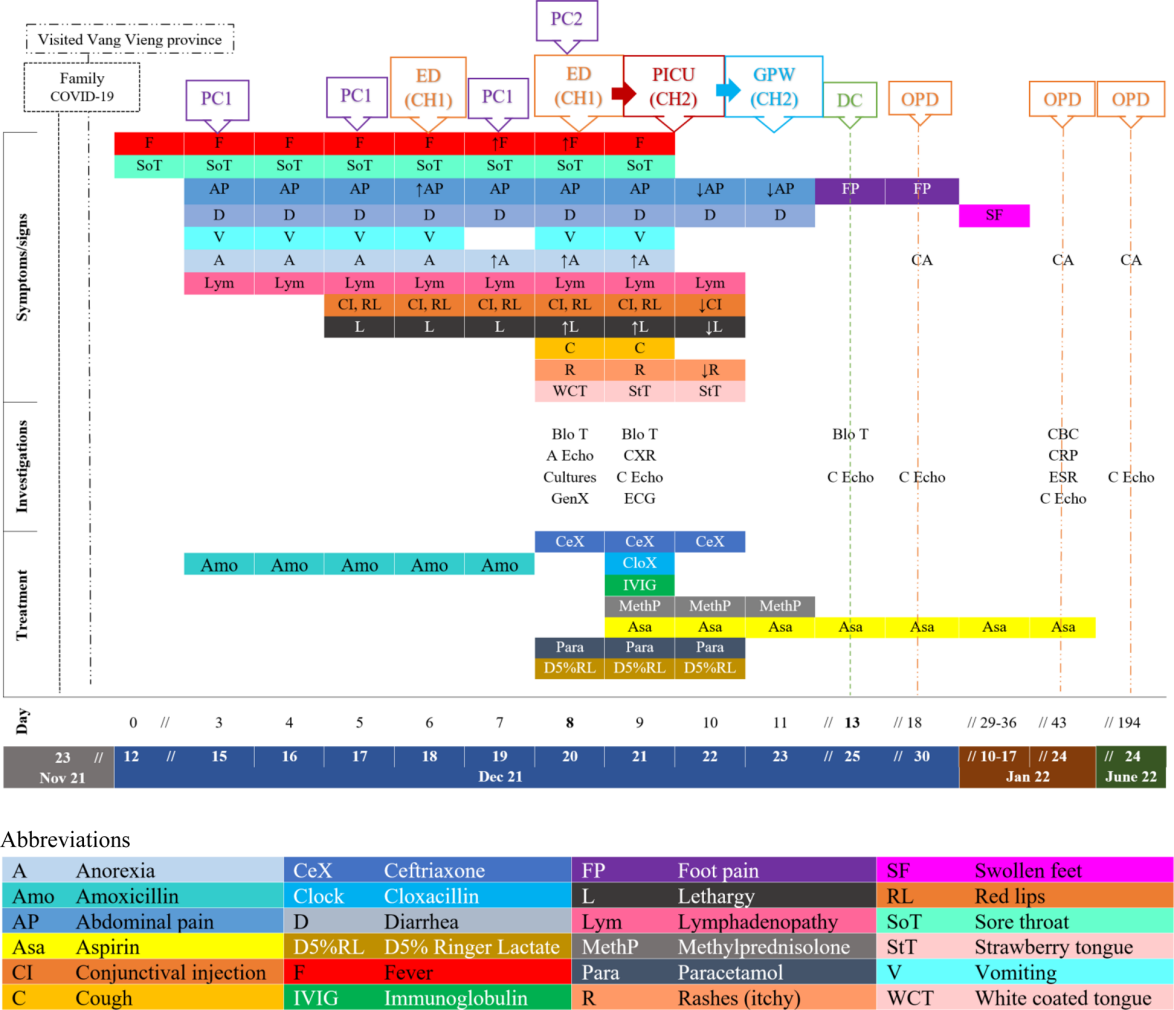
Timeline (dates and days of illnesses) with patient's clinical symptoms and signs in relation to health care services sought, investigations, treatment, and outcomes since the period of possible COVID-19 exposure, to the day of symptoms onset (12 December 2021), the discharged date (25 December 2021), and three follow-up dates (30 Dec 21, 24 Jan, and 24 June 22). (A Echo = Abdominal echography, Blo T = blood test, CA = Clinical assessment, C Echo = Cardiac echography, CH = Central hospital, CBC = complete blood counts, CXR = Chest x-ray, D = discharged, ECG = electrocardiogram, ED = Emergency department, ESR = Erythrocyte sedimentation rate, GPW = General Pediatric Ward, GenX = GeneXpert COVID-19, OPD = Outpatient department, PC = private clinic, PICU = Pediatric Intensive Care, PIDW = Pediatric infectious disease ward, ↑/↓ = Increasing/decreasing, → = Transfer/admit).

His first symptom (day 0) was an intermittent “high grade” fever (the mother did not measure his temperature) associated with chills and daily intermittent mild sore throat, relieved with paracetamol. On days 2–4, he developed mild intermittent periumbilical cramping pain and yellow watery stools without melena/hematochezia which progressed to vomiting with anorexia. He was found to have a 1–2 cm swollen area on his left posterior neck that spread to the right side over 2–3 days. This area was not red nor warm nor tender. His parent brought him to a private clinic (PC1) where he was diagnosed with tonsillitis and given amoxicillin without improvement. On day 5, he was more fatigued and developed bilateral nonexudative conjunctival injections, red lips, and increased anorexia. On day 6, the fever became persistent despite antipyretics. His abdominal pain, vomiting, and diarrhea worsened. His family brought him to an emergency department (ED) at a central hospital (CH1). He was diagnosed with acute gastroenteritis without dehydration and was discharged with oral rehydration solution and analgesia/antipyretics. On day 7, he returned to PC1, with no change in diagnosis nor treatment (timeline in Figure 1).

On the day of admission (day 8), his symptoms had not improved, and his family brought him to a new private clinic (PC2) where he was diagnosed with suspected severe sepsis with concerns for typhoid fever. At PC2, he had lymphopenia of 0.6 × 10^9^/L–5%, granulocytosis of 11.8 × 10^9^/L–91.8%, slightly raised CRP, negative dengue, and scrub typhus antigen testing, as well as a normal abdominal ultrasound. Subsequently, he was referred to the ED. His fever, fatigue, anorexia, and decreased urination continued, but the diarrhea decreased to once a day. He developed an occasional dry cough.

He did not have any flank pain, urinary symptoms, other upper respiratory tract infection symptoms, chest pain, shortness of breath, hypogeusia/ageusia, change in senses of smell/anosmia, altered level of consciousness, headache, dizziness, visual disturbances, focal neurological symptoms, joint/muscle/bone pain or swelling, rash/eschar.

His mother and youngest sister had positive PCR results for COVID-19 three weeks prior. During that time, he had a low-grade fever and mild respiratory symptoms (runny nose and cough), but he was not tested for COVID-19. Two weeks prior to his illness, he travelled to Vang Vieng, a vacation area, where he swam in a shallow river. The family denied other recent travel.

The patient was previously healthy with normal development. He had completed the basic Lao national immunization schedule but had no COVID-19 immunization. He did not take any regular medications and had no known allergies, nor surgical history. There was no family history of allergy and auto-immune diseases. Based on his family's report of the average nominal monthly consumption per capita (KIP) (13), the child's family wealth quintile was reported to be at the 5th quintile (most wealthy).

### Clinical findings

**Figure 2:**
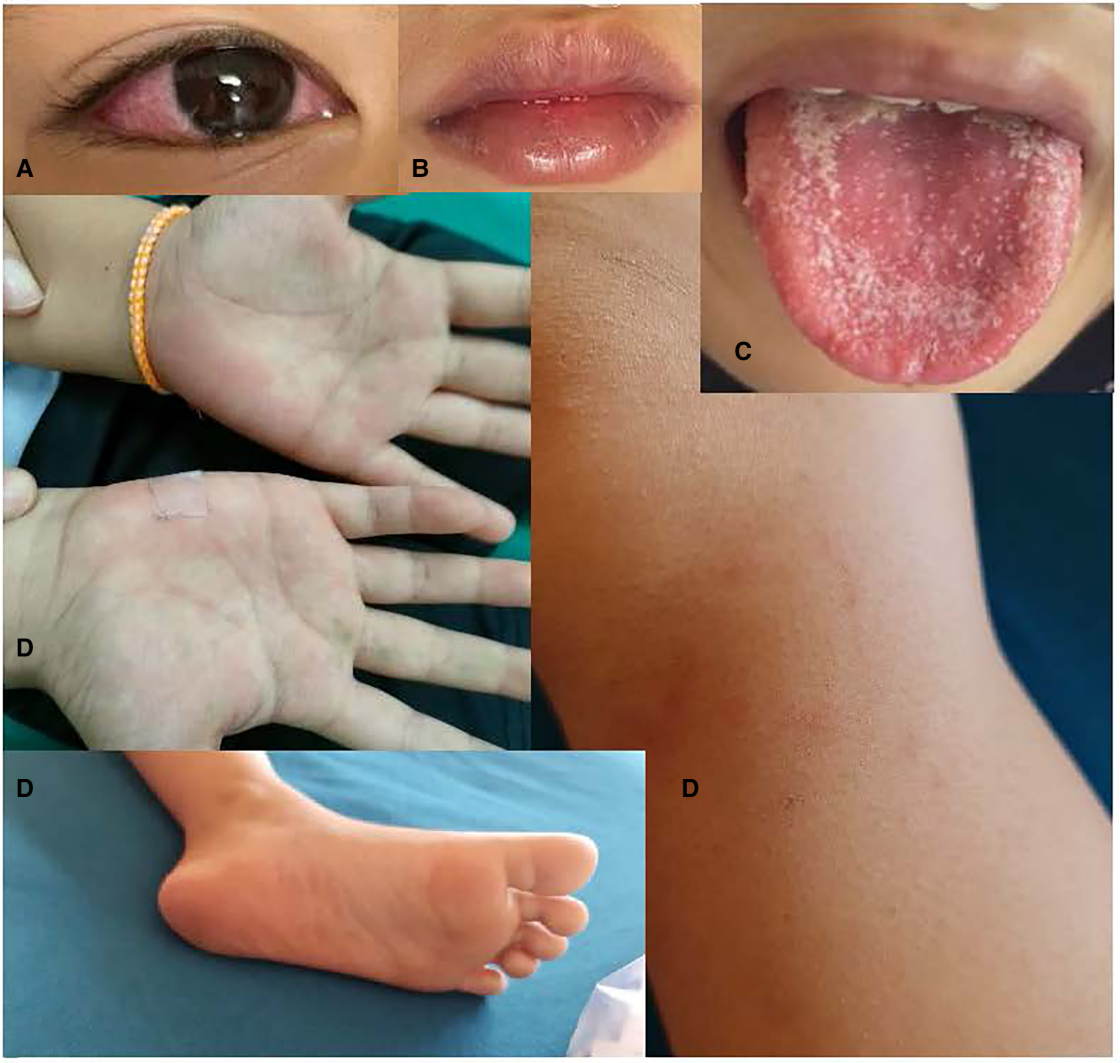
(**A**) Non-exudative conjunctival infection, (**B**) red lips, (**C**) strawberry tongue (onset day 9: 21/12/21), (**D**) itchy pink branching maculopapular rashes on palms, feet & left knee.

At admission his vital signs were: temperature = 39.4 °C; respiratory rate = 50 bpm, heart rate = 150 bpm (regular), SpO2 = 95% on room air. His blood pressure was not recorded. His weight was 28.3 kg (weight for age = −0.4 SD) and height was 124 cm (height for age = −1.9 SD). The patient was fatigued, but alert, orientated and cooperative. He had warm pink skin, capillary refill of < 2 s, and normal skin turgor. He had non-exudative conjunctival injection, red full lips, a white coating on his tongue, and itchy pink blanching maculopapular rashes at his palms, soles, and left knee (Figure 2). Non-tender smooth mobile enlarged lymph nodes were present in the bilateral posterior cervical chain (1.5–2 cm) and bilateral posterior triangles (left: 2 × 3 cm, right: 1.5–2 cm). There were no eschars, bruises, petechiae, desquamations, calf/muscle/joint/bone pain nor edema.

He had mild intercostal retractions. His apical impulse was palpated at the 4–5th intercostal space in the midclavicular line. He had normal S1 and S2, but with reduced heart sounds. We heard no murmur nor additional heart sounds. His lung sounds were normal. He had mild abdominal distension with generalized tenderness on palpation, especially in the right upper quadrant with mild to moderate guarding. There was no rebound, percussion, nor flank tenderness. Bowel sounds were normal with no hepatosplenomegaly. The genital examination was not remarkable. He had no meningismus and the neurological examination was grossly normal.

### Diagnostic assessment

Based on the history and findings on the physical examination, MIS-C was thought to be the likely diagnosis. Cultures (blood, throat swab, urine, and stool) were sent (see Figure 1) and returned negative. COVID-19 rapid test and COVID-19 GeneXpert were also negative. Within three hours of admission, the patient's mental status changed from “A (alert)” to “V (responsive to voice)”. He maintained his blood pressure at 90/60 and was able to walk to the toilet to pass urine with minimal assistance. He had a high fever (39–40 °C), tachycardia (150s), tachypnea (50–58), and desaturated (93% on room air) requiring oxygen (1-liter per minute) by nasal cannula to maintain his SpO2 at 97%. The patient was admitted late in the evening therefore, inflammatory markers (CRP, ESR, ferritin, LDH, albumin), liver and renal function tests, chest x-ray (CXR), electrocardiogram (ECG) and echocardiogram were unavailable and planned to be carried out after transfer and consultation with the pediatric cardiologist at CH2. Upon admission to CH2, COVID-19 RT-PCR was again negative. CXR showed mild cardiomegaly with normal lung fields. The initial echocardiography revealed mild impaired left ventricular function (ejection fraction of 45%–51%) with no dilatation of coronary arteries. His electrocardiogram was normal. In our region, dengue and scrub typhus are common and antigen testing for these illnesses were done and were negative. His urine analysis was unremarkable. The COVID-19 IgG was tested later as an outpatient in follow up and was found to be positive (Figure 1).

### Therapeutic interventions

The patient had previously received five days of amoxicillin without improvement. On day 9 of illness, he clinically worsened and the following were started: intravenous immunoglobulin (IVIG at 2 g/kg in a single infusion over 12 h), methylprednisolone (2 mg/kg/day for 3 consecutive days), aspirin (3.5 mg/kg/day) ceftriaxone (100 mg/kg/day) for four total doses and cloxacillin (50 mg/kg/dose) for one dose. Cloxacillin was started for concern for toxic shock syndrome and stopped as patient stabilized. Rest, oral nutrition, and oral hydration were also encouraged.

### Follow-up and outcomes

**Figure 3:**
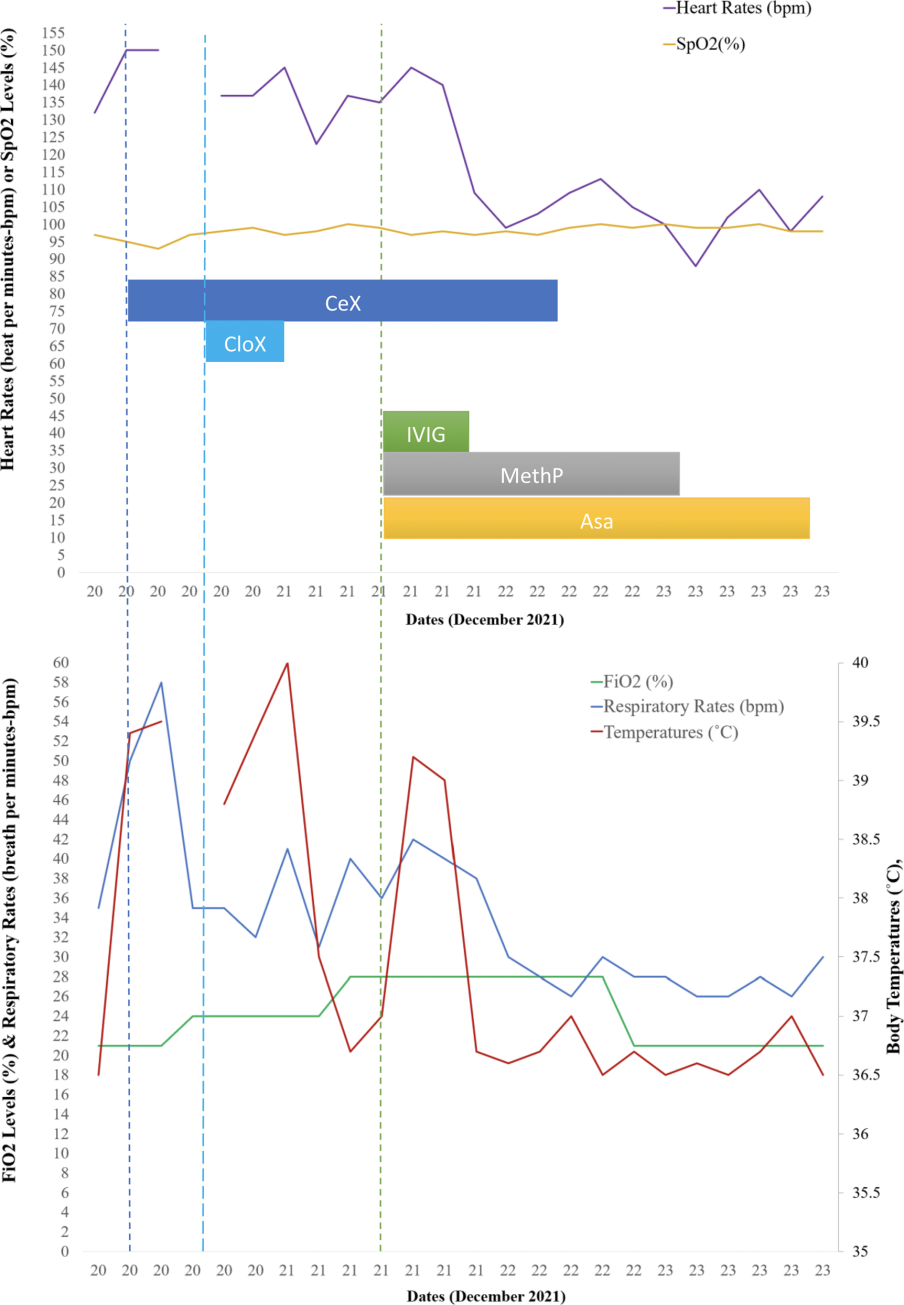
Cardiovascular and respiratory situations in relation to pharmacological therapy.

Twenty-four to 48 h after initiating IVIG and methylprednisolone, his clinical symptoms and fever improved. His heart and respiratory rates normalized, and oxygen therapy was no longer required (Figure 3). The lymphopenia and mild impaired cardiac function resolved (Figure 1). He was discharged from the hospital after 5 days with aspirin. He reported mild bilateral knee pain without swelling, warmth nor redness on the date of discharge, lasting for approximately one week. He later developed bilateral feet swelling for one week, which also spontaneously resolved. On the second follow-up visit, complete blood counts (CBC) and CRP had completely normalized, while ESR significantly improved from ten times upper normal limit to five times the upper normal limit. During these visits, he had repeated echocardiograms which were normal (Figure 1).

### Health care expenses

The patient's family did not have health insurance, which is available in Lao PDR. The family paid around US$ 1,185 for the management of the patient during hospital admissions and the follow-up visits (July 6, 2022: US$ 1 = 17,750 Lao kips), which consisted of services/equipment (6%), medications (86%), and investigations (8%). The IVIG and methylprednisolone costs were around US$ 949 (∼US$15 per gram) and US$ 42 respectively, which constituted about 80% and 3.6% of the total treatment cost. The price the family paid at the government hospital is equivalent to the cost of the service, however, the price at the private clinics is higher. At the time of his hospitalization the test for IgG for SARS-CoV-2 was not available in the public hospital and this needed to be sent to a private laboratory which was the most expensive investigation costing US$29 (2% of total treatment cost).

## Discussion

This is the first ever known MIS-C case report in Lao PDR with moderate to severe symptoms in a previously healthy 9-year-old boy. The diagnosis was based on the epidemiologic link to COVID-19 infection (8). Later, serology testing supported this diagnosis. This case report raised many important areas for consideration: delayed diagnosis, importance of ruling out other causes, diagnostic challenges associated with health care access and low-resources.

This child's diagnosis was made on day 8 of his illness after visiting two private clinics, and one ED twice. His delayed diagnosis unnecessarily increased his exposure to antibiotic therapy at the beginning of his illness. This is concerning as antimicrobial resistance is accelerated by the overuse of antibiotics and causes severe infections, complications, longer hospital stays, and increased mortality (14).

Though early recognition and diagnosis of MIS-C are necessary, it is also important to rule out other causes. The patient was first diagnosed and treated for tonsillitis, gastroenteritis, and a suspected typhoid fever bacteremia. At presentation to the hospital alternative diagnoses to MIS-C included Kawasaki disease, bacterial sepsis, staphylococcal toxic shock syndrome, dengue fever, typhoid fever, and severe acute COVID-19.

Though the patient presented with moderate MIS-C symptoms, compared to higher-resourced settings very limited investigations were performed initially. D-dimer, fibrinogen, procalcitonin, interleukin-6, coagulation studies, and cardiac markers were not carried out. Currently all these tests are available at higher prices in private clinics in Lao PDR. At the public hospitals D-dimer, fibrinogen, coagulation studies and cardiac markers are available before 10 am Monday through Friday. While it is well known that suboptimal health care access can lead to diagnostic and treatment delay, worsening disease, and higher disease complications (15), the speed with which the investigations were completed is faster in Vientiane Capital than in most rural areas, while still slower than in many high resource areas. In our opinion, the most useful laboratory tests in this case include full blood counts (FBC), quantitative CRP or ESR, liver function tests (LFTs), dengue rapid diagnosis test (Den RDTs), scrub typhus test, SARS-CoV-2 tests, and cultures. Raised inflammatory markers tend to be associated with illness severity (8); this could potentially assist in the decision to transfer to a more resourced health care facility.

In rural Lao PDR physicians may have access to FBC, LFTs, Den RDT, scrub typhus test, and SARS-CoV-2 RDTs. They will likely have less access to quantitative CRP and ESR, SARS-CoV-2 GeneXpert and PCR, or cultures. Tests such as a procalcitonin or interleukin-6 are not available. Therefore, in these settings the decisions to start, stop, or complete a course of antibiotics must include an assessment of likeliness of MIS-C and the inability to rule out alternative diagnoses.

The most expensive investigation for MIS-C was IgG SARS-CoV-2, followed by ferritin level and cultures. Son (8) suggested that all suspected MIS-C cases be tested for SARS-CoV-2 serology and reverse transcription PCR (RT-PCR). SARS-CoV-2 GeneXpert is no longer available in Lao PDR. Development of lower cost serology and PCR testing would facilitate faster diagnoses in RLS. Son additionally recommends that blood, urine, throat, stool cultures, respiratory viral panel, Epstein-Barr virus serology and PCR, cytomegalovirus serology and PCR, Enterovirus PCR, and Adenovirus PCR should all be carried out for moderate to severe MIS-C (8). Even in our central teaching hospitals, the last five could not be carried out. While it is important to rule out other diagnoses, it may be most prudent to focus on diseases with high prevalence locally with locally affordable and accurate testing. Investing in accurate low-cost testing for disease with high local prevalence will help facilitate faster diagnoses in general, as well as for MIS-C. Inflammatory markers, such as ferritin, are also nonspecific and therefore not testing all the inflammatory markers may be a means for cost savings.

As in most RLS, it was impossible to rule out all alternative diagnoses and therefore it was also important that we monitored this child for response to antibiotics and provided antibiotics that appropriately treated the bacterial infections which were alternative diagnoses while awaiting culture results. Though we did not complete the antibiotic course of the bacterial infections, it may be prudent to complete antibiotic treatment for common illnesses that may be endemic in each area that cannot be satisfactorily ruled out with the available laboratory testing.

CXR and echocardiograms also may not be available in remote settings. Echocardiograms require equipment and experts to perform, however, it is particularly useful to assess and predict levels of MIS-C severity as well as to follow up the cardiac outcomes. The ability to get patients in low and middle-income countries (LMICs) urgent quality echocardiographic evaluation remains difficult in most areas (16).

There was a significant expense related to the management of this patient's MIS-C. This family paid more for treatment than would be possible for most families in Lao PDR. IVIG was the most expensive single item in this patient's care and in Lao PDR it was only available in CH2 and would not be affordable for many patients in LMICs (10). The limited availability and cost of immunomodulatory drugs likely affects the outcome of MIS-C in RLS (12). Currently there is inadequate evidence showing differences in the efficacy and safety of glucocorticoid or IVIG vs. combination treatment (17), international recommendations tend to recommend IVIG or IVIG plus glucocorticoids in the setting of hypotension, unless IVIG is not available or contraindicated (8, 18). When IVIG is unavailable, the first line treatment with pulse methylprednisolone was associated with favorable immediate and short term follow up outcomes (19). This led to considerable deliberation in our circumstance when IVIG is technically available but is cost prohibitive. Fortunately, the parents' financial status facilitated getting this patient all recommended treatment including IVIG, methylprednisolone, and antibiotics. Additionally, the family was able to return for additional investigations, including the costly SARS-CoV-2 IgG and completed all follow-up.

Additionally, at the time of admission to the CH1, there was limited pediatric intensive care unit beds, therefore the patient was transferred to CH2. Many MIS-C patients required ICU admission for cardiac or respiratory support (9). There are many places in Laos where an intensive care bed with appropriate staffing would not be available in a timely manner.

Regardless of the observed low incidence of MIS-C, caution is necessary and pediatric incidence rates of MIS-C and COVID-19 should continue to be monitored aggressively (9). The country's COVID-19 surveillance should be continued, and MIS-C surveillance systems should be established to monitor the impact of COVID-19 on the health of Lao children. As demonstrated above, due to the various clinical presentations, essential emergency/critical care, and possible fatal complications, it is essential to raise MIS-C awareness (10). Improving ability to appropriately identify, rule out, and treat diseases that can present similarly and with similar severity regardless of the patients' location is essential in improving the equity of care for children around the world and in RLS. Lastly, expanded access to a SARS-CoV-2's vaccine for children may be the best solution for decreasing this deadly illness.

## Data Availability

The original contributions presented in the study are included in the article/Supplementary Material, further inquiries can be directed to the corresponding author/s.
